# *Aspergillus korhogoensis*, a Novel Aflatoxin Producing Species from the Côte d’Ivoire

**DOI:** 10.3390/toxins9110353

**Published:** 2017-10-31

**Authors:** Amaranta Carvajal-Campos, Ama Lethicia Manizan, Souria Tadrist, David Koffi Akaki, Rose Koffi-Nevry, Geromy G. Moore, Stephen O. Fapohunda, Sylviane Bailly, Didier Montet, Isabelle P. Oswald, Sophie Lorber, Catherine Brabet, Olivier Puel

**Affiliations:** 1 Toxalim (Research Centre in Food Toxicology), Université de Toulouse, INRA, ENVT, INP-Purpan, UPS, 31027 Toulouse, France; a.carvajalcampos@gmail.com (A.C.-C.); souria.tadrist@inra.fr (S.T.); sylvianebailly7@gmail.com (S.B.); isabelle.oswald@inra.fr (I.P.O.); sophie.lorber@inra.fr (S.L.); 2 Laboratoire de Biotechnologie et Microbiologie des Aliments, UFR des Sciences et Technologie des Aliments, Université Nangui Abrogoua, 02 BP 801 Abidjan, Côte d’Ivoire; manizanlethicia@yahoo.fr (A.L.M.); rosenevry2@gmail.com (R.K.-N.); 3 Laboratoire des Procédés Industriels de Synthèse, de l’Environnement et des Energies Nouvelles, Département Génie Chimique et Agro-alimentaire, Institut National Polytechnique Félix Houphouët-Boigny, BP 1313 Yamoussoukro, Côte d’Ivoire; davidakaki@yahoo.fr; 4 Southern Regional Research Center, Agricultural Research Service, United States Department of Agriculture, New Orleans, LA 70179, USA; geromy.moore@ars.usda.gov; 5 Department of Microbiology, Babcock University, 121003 Ilishan Remo, Nigeria; oystak@yahoo.co.uk; 6 Centre de Coopération Internationale en Recherche Agronomique pour le Développement (CIRAD)—Département PERSYST—UMR QualiSud, 34398 Montpellier, France; didier.montet@cirad.fr (D.M.); catherine.brabet@cirad.fr (C.B.)

**Keywords:** *Aspergillus* section *Flavi*, aflatoxins, cyclopiazonic acid, polyphasic approach, versicolorins

## Abstract

Several strains of a new aflatoxigenic species of *Aspergillus*, *A. korhogoensis*, were isolated in the course of a screening study involving species from section *Flavi* found contaminating peanuts (*Arachis hypogaea*) and peanut paste in the Côte d’Ivoire. Based on examination of four isolates, this new species is described using a polyphasic approach. A concatenated alignment comprised of nine genes (*ITS*, *benA*, *cmdA*, *mcm7*, *amdS, rpb1*, *preB*, *ppgA*, and *preA*) was subjected to phylogenetic analysis, and resulted in all four strains being inferred as a distinct clade. Characterization of mating type for each strain revealed *A. korhogoensis* as a heterothallic species, since three isolates exhibited a singular *MAT1-1* locus and one isolate exhibited a singular *MAT1-2* locus. Morphological and physiological characterizations were also performed based on their growth on various types of media. Their respective extrolite profiles were characterized using LC/HRMS, and showed that this new species is capable of producing B- and G-aflatoxins, aspergillic acid, cyclopiazonic acid, aflavarins, and asparasones, as well as other metabolites. Altogether, our results confirm the monophyly of *A. korhogoensis*, and strengthen its position in the *A. flavus* clade, as the sister taxon of *A. parvisclerotigenus*.

## 1. Introduction

The presence of mycotoxins in agricultural commodities poses serious economic and health risks [1,2,3]. Among the mycotoxins, aflatoxins are by far the most studied since their ingestion can cause deleterious health effects in humans and animals including hepatic cancer and, in some instances, death [4]. Aflatoxin B_1_ is the potent compound of this chemical family as it displays mutagenic, teratogenic and hepatocarcinogenic effects in humans and animals [5]. To date, it is considered as the most carcinogenic, teratogenic and genotoxic substance of natural origin [6,7]. 

Species that have so far been reported to produce aflatoxins are all classified in *Aspergilllus* subgenus *Circumdati* and section *Flavi*, with the exception of two other species originally sampled in the Côte d’Ivoire, *A. ochraceoroseus* and *A. rambelli* [8,9]. Species from *Aspergillus* section *Flavi* represent a well-known group of saprophytic filamentous fungi, several of which have the ability to produce beneficial secondary metabolites or enzymes used in food fermentation and biotechnology, such as kojic acid and α-amylase [10]. Conversely, some of these species have the potential to produce one or more harmful mycotoxins, such as aflatoxins, cyclopiazonic acid, versicolorins, and aflatrems [11,12]. Due to extensive research into their aflatoxin production, *A. flavus*, *A. parasiticus*, and *A. nomius* are considered major species in section *Flavi*. Among them, *A. flavus* is the most important because of its worldwide distribution, and it represents the largest source of aflatoxin B_1_ contamination of several staple crops, including maize, tree nuts, peanuts, cottonseed, grains, cassava, and spices [13,14,15,16]. 

Although some species relationships in section *Flavi* are still unclear, the section can be separated into one of seven main clades based on a polyphasic approach: *A. flavus* clade, *A. parasiticus* clade, *A. tamarii* clade, *A. nomius* clade, *A. alliaceus* clade, *A. togoensis* clade, or *A. avenaceus* clade, and *A. mottae* and *A. bertholletius* [17,18,19]. Each clade may contain cryptic species that are difficult to identify, based solely on morphological characters or extrolite profiles, but can be delineated using a polyphasic approach that also includes molecular analyses [12,17,18]. It has been suggested that two cryptic species, *A. minisclerotigenes* and *A. parvisclerotigenus*, belong to the *A. flavus* clade [9,20]. However, in addition to B-aflatoxins these species produce G-aflatoxins, which *A. flavus* is incapable of producing [17,21] due to a deletion of genomic sequence between the aflatoxin pathway genes, *norB* (*aflF*) and *cypA* (*aflU*), thereby altering the promoter and coding regions [22].

In this paper, we describe *A. korhogoensis* sp. nov. as a novel cryptic species within the *A. flavus* clade, based on a polyphasic analysis of four strains isolated from peanuts collected in the region of Korhogo, Côte d’Ivoire. 

## 2. Results

### 2.1. Molecular Analyses

#### 2.1.1. Multilocus Phylogenetic Analysis

**Figure 1:**
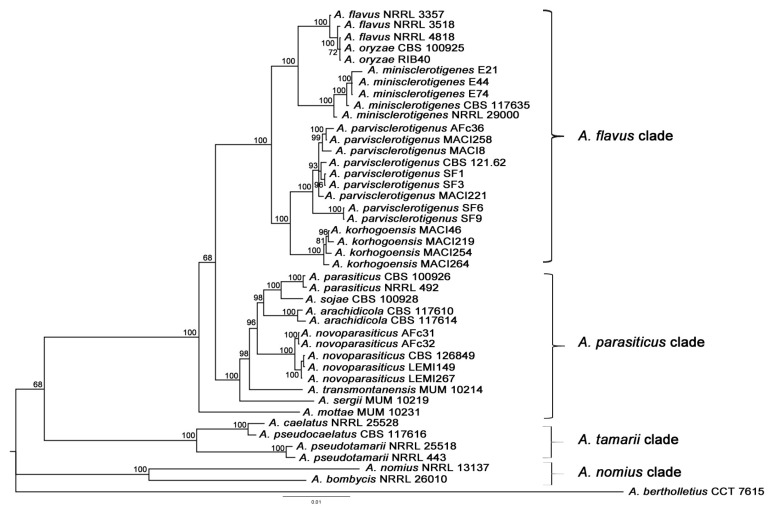
Phylogenetic tree of *Aspergillus* section *Flavi* based on concatenated sequences from nine genomic loci (*ITS*, *benA*, *cmdA*, *mcm7*, *amdS*, *rpb1*, *preB*, *ppgA*, and *preA*). Bayesian tree was calculated from 41 strains, and includes the Type strain for most species. Strong bootstrap values are shown at branch nodes. Species isolate numbers are indicated at branch tips. *A. bertholletius* CCT 7615 was used as the outgroup taxon.

The phylogenetic tree inferred from nine concatenated genes (*ITS*, *benA*, *cmdA*, *mcm7*, *amdS*, *rpb1*, *preB*, *ppgA*, and *preA*), obtained from Bayesian and ML analyses, yielded largely similar topologies, particularly congruent for *Aspergillus flavus* clade. Here, we chose the Bayesian topology as hypothesis of phylogenetic relationships because the results were of greater robustness (Figure 1). Our results support previous phylogenetic inferences involving species from section *Flavi*. *Aspergillus bertholletius* was used as the outgroup taxon. The *A. nomius* clade, which included *A. bombycis* and *A. nomius*, was monophyletic and appeared as a basal group (Posterior Probability, PP = 1). The topology then split in two robust groups, one formed by the *A. tamarii* clade, which included *A. caelatus*, *A. pseudocaelatus* and *A. pseudotamarii*, and a second monophyletic group, which included the *A. parasiticus* and *A. flavus* clades, as well as *A. mottae*. This latter was placed as the ancestral taxon of the group including *A. parasiticus* and *A. flavus* clades. *Aspergillus parasiticus* clade was consistent with Soares et al. results [18] and included *A. parasiticus*, *A. sojae*, *A. arachidicola*, *A. novoparasiticus*, *A. sergii* and *A. transmontanensis*. *A. sergii* and *A. transmontanensis* are basal taxa, respectively. The *A. flavus* clade included *A. flavus*, *A. oryzae*, *A. minisclerotigenes*, *A. parvisclerotigenus* and *A. korhogoensis*, the herein described new species.

The *A. flavus* clade is comprised of two main groups: one that includes *A. flavus*, its domesticated species *A. oryzae* and *A. minisclerotigenes* (PP = 1); and the other group encompasses *A. parvisclerotigenus* and *A. korhogoensis* sp. nov. (PP = 1). The four isolates (MACI46, MACI219, MACI254 and MACI264) putatively identified as *A. korhogoensis* were tightly clustered, suggesting they were a distinct species from *A. parvisclerotigenus*. Strains from the latter species included isolates from different populations, which were clustered together, suggesting a monophyletic group with no major differences among populations.

*Aspergillus novoparasiticus* was represented by two well-supported groups, which segregated based on geography since one group corresponds to the strains isolated from Brazil [23], while the other group corresponds to strains isolated from Benin [14]. Besides their geographical distributions, both groups were isolated from different environments, such as hospital environments (Brazil samples) or foodstuffs (Benin samples).

#### 2.1.2. Mating Type Analysis

Results from our mating type diagnostic PCR revealed that isolates MACI46, MACI254 and MACI264 contained a single *Mat1-1* gene, and that isolate MACI219 contained a single *Mat1-2* gene. These findings demonstrate that *A. korhogoensis* sp. nov. is likely a heterothallic (self-infertile) fungus. Whether these mating-type genes are functional is unknown. Future mating tests will be required to determine this.

**Figure 2:**
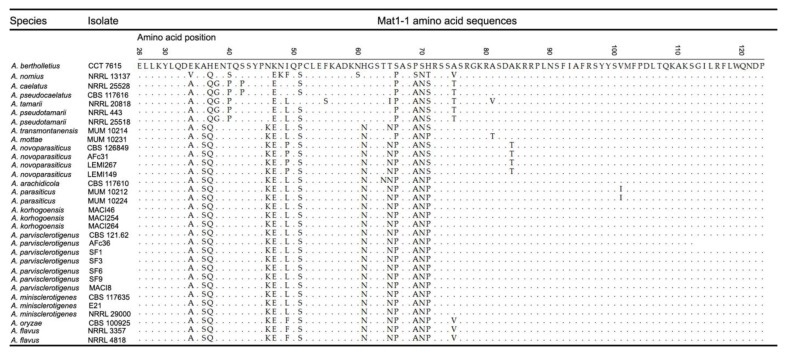
Amino acid sequence alignment for the *Mat1-1* locus in examined strains representing several *Aspergillus* species. The amino acid positions were determined based on the complete amino acid sequence of *A. flavus* NRRL 3357 strain (accession number = EED46656). Accession numbers recovered from GenBank: *A. parasiticus* MUM 10.212 = HM80303, *A. parasiticus* MUM 10.224 = HM803058, and *A. tamarii* NRRL 20818 = HM803044. Other accession numbers are given in Table S2.

The MAT1-1 amino acid sequence of *A. bertholletius* was used as the reference sequence to compare with *Mat1-1* genes from other examined taxa. Basal taxa (*A. bertholletius*, *A. nomius*, *A. caelatus*, *A. pseudocaelatus*, *A. tamarii* and *A. pseudotamarii*) presented alanine, asparagine, histidine and threonine at position 36, 46, 61 and 65, respectively, which changed in derived species into serine, lysine, asparagine and asparagine (Figure 2). The *A. tamarii* clade presented four apomorphies that are specific to the clade, and one apomorphy that was fixed in the derived species. The *A. parasiticus* and *A. flavus* clades exhibited a highly conserved MAT1-1 amino acid sequence, except for one amino acid substitution in *A. parasiticus* at position 101, and two substitutions in both *A. flavus* and *A. oryzae* at positions 49 and 75. Although haplotypes of *A. minisclerotigenes*, *A. parvisclerotigenus* and *A. korhogoensis* sp. nov. shared identity for their respective MAT1-1 amino acid sequences (Figure 2), there were single nucleotide polymorphism (SNP) differences that did not result in an amino acid replacement. *Aspergillus minisclerotigenes* exhibited two apomorphies, whereas *A. parvislerotigenus* and *A. korhogoensis* sp. nov. each exhibited only one (except MACI264, which exhibited the conserved ancestral state).

**Figure 3:**
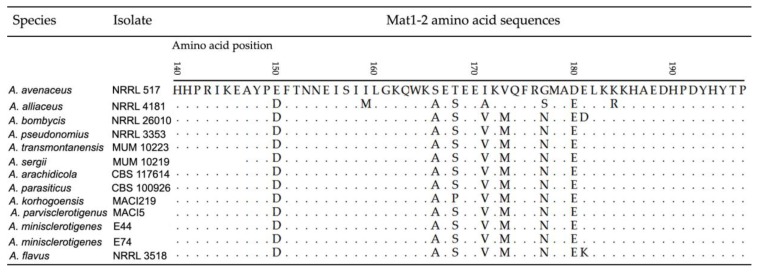
Amino acid sequence alignment for the *Mat1-2* locus in examined strains representing several *Aspergillus* species. The amino acid positions were determined based on the complete amino acid sequence of *A. bombycis* NRRL 26010 strain (accession number = OGM45987). Accession numbers recovered from GenBank: *A. avenaceus* NRRL 517 = HM802955, *A. alliaceus* NRRL 4181 = HM802964, and *A. transmontanensis* MUM 10223 = HM802958. Other accession numbers are given in Table S2 except for *Aspergillus parvisclerotigenus* MACI5 (MF966968). *Aspergillus parvisclerotigenus* MACI5 species identification was based on genomic sequences from ITS (KY689161), *benA* (KY628772) and *cmdA* (KY661269). The *A. sergii* sequence available from GenBank is shorter.

In the case of *Mat1-2* gene, amino acid sequences were identical for *A. nomius*, *A. pseudonomius*, *A. sergii*, *A. transmontanensis*, *A. arachidicola*, *A. parasiticus*, *A. parvisclerotigenus* and *A. minisclerotigenes*. There was one substitution in the amino acid sequence for *A. korhogoensis* sp. nov. (S168P), and one for *A. flavus* (E181K) (Figure 3). Basal taxa, *A. avenaceus* and *A. alliaceus*, exhibited several differences in their amino acid sequences.

### 2.2. Secondary Metabolism Characterization

An analysis of secondary metabolites produced by the four *A. korhogoensis* strains was performed and the results are summarized in Table 1. Metabolites were identified according to the Metabolomics Standard Initiative level definitions [24]. Metabolites were identified at level 1 when they displayed the same retention time, and UV and MS/MS spectra as the authentic standard. They were identified at level 2 when the metabolites shared the same UV spectrum and/or the same MS/MS fragmentation pattern in accordance with the literature.

All strains produced aflatoxins B_1_, B_2_, G_1_, and G_2_, as well as several aflatoxin biosynthetic intermediates including 3-*O*-methylsterigmatocystin, sterigmatocystin and versicolorins A and B. Cyclopiazonic acid (CPA) and different other members of the CPA family were detected in the whole fungal extract of each strain. This new species also produced kojic acid, aflatrem, and its precursors or related compounds (paspaline, paspalinine, paxilline, and 13-desoxypaxilline). Aflatrem and paspaline were also present in the sclerotium extracts, as well as another related compound that appears to be an uncharacterized hydroxylated form of aflatrem ([M + H]^+^*/z* = 518.28992; deviation = −0.347 ppm) previously evoked by Nicholson et al. [33]. Leporin B and its precursor were previously detected in culture extracts [25]. 

The HPLC-DAD analysis of sclerotium extracts revealed the presence of members of at least three families. First, five compounds showed a typical anthraquinone UV spectrum (nm): 223 (100%), 269sh, 293, 319sh, 455. On the bases of UV and MS/MS fragmentation patterns, this compound was identified as asparasone A [31,34]. Three other asparasone-derived anthraquinones were identified in sclerotium extracts (Table 1). Six compounds displayed an aflavarin UV spectrum (nm): 221 (100%), 238sh, 291, 310, 322sh [35]. The LC-HRMS allowed the identification of aflavarin and four aflavarin-associated compounds previously reported by Cary et al. [32]. The last chemical family identified in *A. korhogoensis* sclerotia were aflavinines [36,37]. Indeed, three compounds with a typical aflavinine UV spectrum (nm): 224 (100%), 283, and 291, were present in the sclerotium extracts of each tested strain. The comparison with the 20-hydroxyaflavinine standard confirmed the presence of this metabolite.

### 2.3. Taxonomy

**Figure 4:**
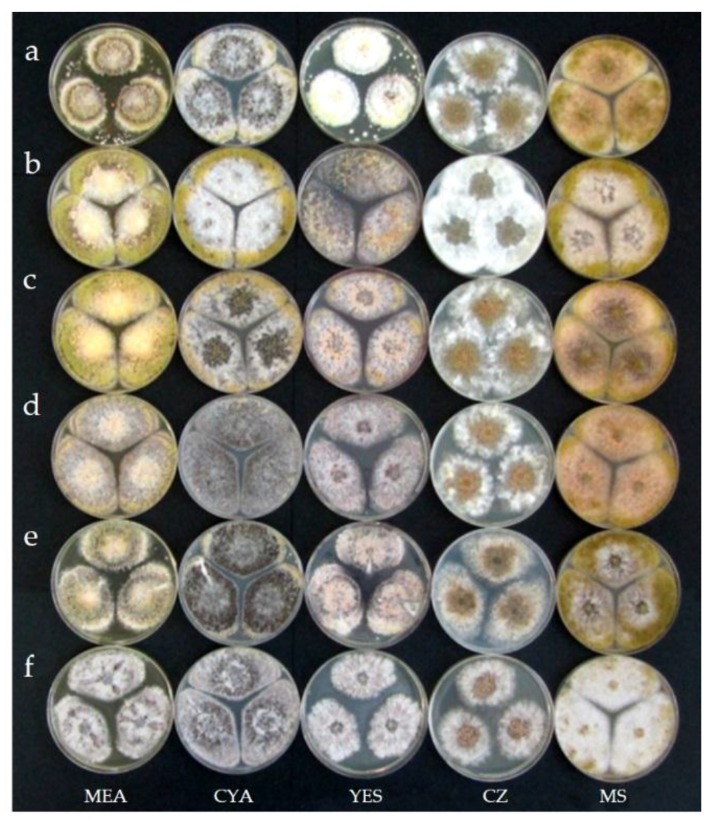
Comparison between cultures of *Aspergillus korhogoensis* sp. nov. and other species from the *A. flavus* clade: (**a**) *A. korhogoensis* MACI46; (**b**) *A. korhogoensis* MACI219; (**c**) *A. korhogoensis* MACI254; (**d**) *A. korhogoensis* MACI264; (**e**) *A. parvisclerotigenus* CBS 121.62; and (**f**) *A. minisclerotigenes* J117c. Cultures were grown on MEA, CYA, YES, CZ, and MS at 25 °C for Seven days.

**Figure 5:**
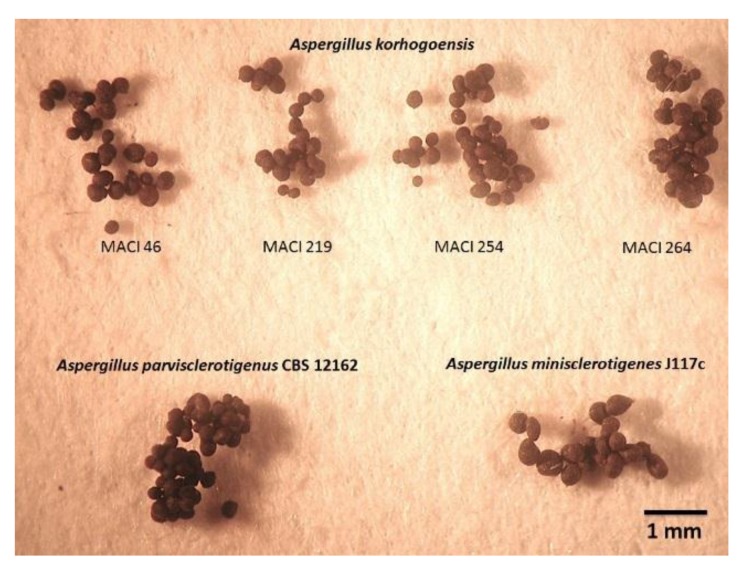
Comparison between sclerotia of *A. korhogoensis* sp. nov. and other species from *A. flavus* clade. Sclerotia recovered from cultures grown on MEA at 25 °C for seven days.

**Figure 6:**
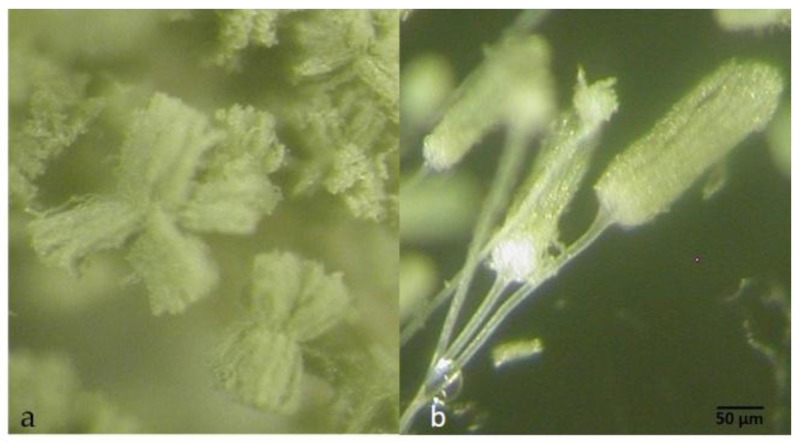
Conidial heads of *A. korhogoensis* MACI254 (100×): (**a**) radiate splitting conidial heads; and (**b**) columnar conidial heads (100×).

**Figure 7:**
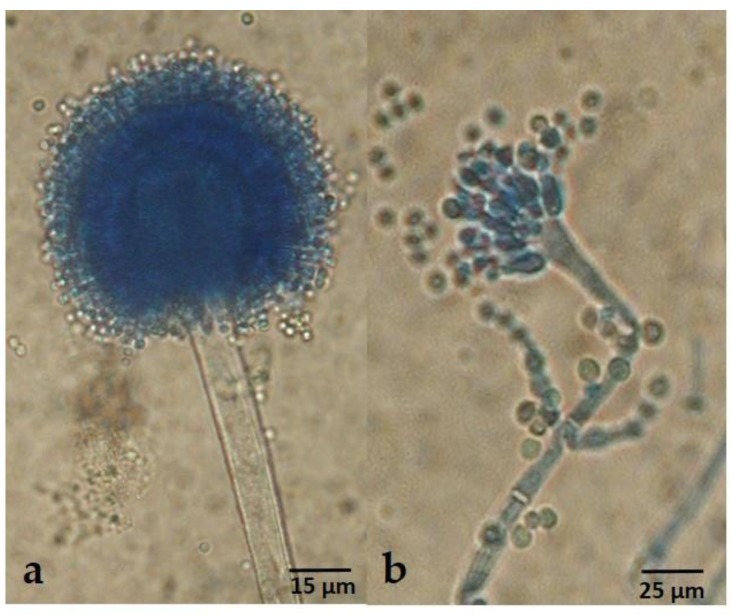
Conidiophores of *A. korhogoensis* MACI254 (400×): (**a**) typical conidiophore, radiate and biseriate, mostly observed in basal mycelium; and (**b**) atypical conidiophore uniseriate, found in aerial mycelium.

***Aspergillus korhogoensis*** A. Carvajal-Campos, A.L. Manizan, S. Tadrist, D.K. Akaki, R. Koffi-Nevry, G.G. Moore, S.O. Fapohunda, S. Bailly, D. Montet, I.P. Oswald, S. Lorber, C. Brabet and O. Puel sp. nov. (Figure 4, Figure 5, Figure 6 and Figure 7).

***Etymology*:** The specific epithet “*korhogoensis*” is a noun in the genitive case and refers to the Korhogo region located in the Côte d’Ivoire, from where the new species was isolated.

***Diagnosis*:** Colonies on MEA deeply floccose with a dominant white aerial mycelium. Sporulation dull yellowish green. Abundant sclerotia (especially on MEA and CYA), mostly at the colony surface, small size (<400 µm), dark brown at mature state; conspicuous amber exudate produced by sclerotia. Reverse orange to brownish orange, more conspicuous on MS, and on MEA and CYA presence of concentric rings on orange shades. Conidial heads typically radiate, fertile upper 75% of their surface and splitting, less frequent narrow and long columnar to short columnar, rarely micro-heads. Conidiophores of radiate heads are hyaline, long, large and slightly roughened, whereas conidiophores of columnar heads and micro-heads are short, narrow and smooth. Conidial heads biseriate for radiate heads, and uniseriate for the others. Vesicles oblong to spatulate, 25–47 µm in diam; metulae 6.7–11.2 µm X 4–5.5 µm; phialides 7–10 µm X 3–5.7 µm; conidia yellowish green to green, oblong and smooth or slightly rough, 3–5 µm diam. 

***Colony diameters*:** After seven days at 25 °C, colonies reached 37–60 mm on MEA, 59–67 mm on MS, 36–57 mm on YES, and 57–80 mm on CYA. Colonies kept seven days at 37 °C on MEA reached 38–57 mm, whereas colonies kept at 42 °C reached 7.5–12 mm. 

***Physiological studies*:** All strains analyzed on AFPA showed a bright orange reverse, a sign of aspergillic acid production. The colonies did not sporulate and presented reduced aerial mycelia. On CREA, the strains showed a positive production of organic acids, except for MACI219.

***Extrolite production*:** Aflatoxins B_1_, B_2_, G_1_, G_2_, 3-*O*-methylsterigmatocystin, versicolorins A and B, aspergillic acid, α- and β-CPA, 2 oxo-CPA, aflatrem, paspaline, paspalinine, aflavarins, asparasones, aflavinines, leporin B.

*Aspergillus korhogoensis* sp. nov. exhibited phenotypic characters that place it within the *A. flavus* clade, such as conidial heads typically radiate that split into several columns in green shades. Phenotypically, the new species resembled *A. parvisclerotigenus*. Both species shared several common traits, making difficult to distinguish between them; however, some subtle differences were observed. The new species grew faster on MEA and CYA at 25 °C than *A. parvisclerotigenus*, and the reverse coloration on MEA and MS was orange for *A. korhogoensis* and cream for *A. parvisclerotigenus*. The size of sclerotia was also comparatively smaller in *A. korhogoensis*. On MEA at 42 °C, *A. parvisclerotigenus* grew faster (15.5–20 mm) than *A. korhogoensis*, for which growth was reduced (7.5–12 mm) or inexistent in strain MACI219. On AFPA, *A. parvisclerotigenus* colonies were mildly to highly floccose, produced profuse sclerotia and conidia in yellowish shades, whereas *A. korhogoensis* sp. nov. colonies exhibited sparse aerial mycelium and sclerotia, and conidia were almost non-existent. 

***Holotype*:** Isolated from Gbandokaha. Deposited in the NRRL collection.

***Isolates examined*:** MACI254 (NRRL 66710), Côte d’Ivoire, Gbandokaha (9°32′ N, 5°33′ W), from peanut pods, 15 November 2014, A.L. Manizan MACI254. MACI46 (NRRL 66708), Côte d’Ivoire, Korhogo (9°29′ N, 6°49′ W), from peanut seeds, 19 November 2014, A.L. Manizan MACI46. MACI219 (NRRL 66709), Côte d’Ivoire, Pokaha (9°24′ N, 5°30′ W), from peanut pods, 15 November 2014, A.L. Manizan MACI219. MACI264 (NRRL 66711), Côte d’Ivoire, Gbandokaha (9°32′ N, 5°33′ W), from peanut pods, 17 November 2014, A.L. Manizan MACI264.

***Habitat*:** Found on peanuts. 

***Distribution*:** Korhogo region, North Côte d’Ivoire.

## 3. Discussion

The number of species in section *Flavi*, in direct correlation with the number of species capable of producing aflatoxins, has increased over the last decade [17,18,19,23]. Although unable to produce aflatoxins, a 26th species, *A. hancockii* sp. nov., was very recently identified and grouped with section *Flavi* species [38]. The use of a polyphasic approach to characterize a species, based on the unified species concept [39,40], has enabled mycologists to acknowledge cryptic diversity in *Aspergillus* section *Flavi*. By using this approach, morphological, physiological, and molecular characters are integrated to understand species relationships [12,17,18,19,20,23,41]. It is accepted that the use of a single approach will mask the exact relationships among species, not only in Aspergilli, but also in other fungi [12].

In the present study, we included an ensemble of six genomic regions already tested to be informative for section *Flavi* (*ITS*, *benA*, *cmdA*, *mcm7*, *rpb1* and *amdS*). Three other genes (*ppgA*, *preA*, *preB*), reportedly involved in sexual development, were added and the set resulted in a concatenated sequence of 4624 bp. These three genes are required in the specific mating recognition. *PreA* and *preB* are MAT target genes that encode a-pheromone and α-pheromone receptors, respectively [42]. *PpgA* encodes the α-pheromone precursor that binds to PreB [43]. To our knowledge, this is the first study to make a phylogenetic inference that includes genes *PreA*, *PreB* and *PpgA*. Some authors have suggested that the accuracy of the phylogenetic approach could be increased by adding molecular data with different evolutive rates, diminishing possible artifacts caused by polymorphic haplotypes [44,45] and providing more robust information to elucidate potential complexity within a clade. The use of nine concatenated genes resulted in a robust phylogenetic tree topology, which includes the most important species of the section in terms of economic and public health impact, as well as species described in the last decade (not basal taxa). The results of the present study were congruent with several studies performed involving species from section *Flavi* [17,18,19,20,23]. The tree obtained from nine concatenated genes showed a clear partition of *A. novoparasiticus* strains in two, one subgroup containing South American isolates and another containing African isolates. More *A. novoparasiticus* strains isolated from both continents would be needed in order to confirm this observation. Additionally, the ensemble allowed to determine a cryptic species, *A. korhogoensis* sp. nov.

Most species in section *Flavi* are considered heterothallic, containing either the *MAT1-1* or *MAT1-2* idiomorph [15,18,46], and from the present study so is *A. korhogoensis*. Thus far, *A. alliaceus* is the only homothallic species in section *Flavi* [46]. Reportedly, strains of *A. nomius* may contain both idiomorphs, but only one is functional [11]. Diversity from sexual reproduction in these fungi is expected to arise from out-crossing of heterothallic species, between complementary strains that are able to produce sclerotia [47,48]. Laboratory crosses between sexually compatible strains showed that sexual reproduction was possible in *A. parasiticus*, *A. flavus*, and *A. nomius* [11,49,50]. Inter-specific hybridization was shown to be a possibility via laboratory crosses that resulted in viable ascospores, including recombinant offspring, being produced [51]. Moreover, sexual reproduction is more likely to occur within populations having a 1:1 ratio of both idiomorphs [11,42,48], although asexual reproduction is still a large component to the life cycle of micro-fungi such as the Aspergilli [52]. Presence of both idiomorphs in *A. parvisclerotigenus,* and *A. korhogoensis* suggests that cryptic sexuality might occur in natural populations, yet laboratory mating experiments involving these species is necessary to yield conclusions. As well, more population-scale field sampling of strains from these species are necessary to determine if they have a history of recombination as observed in *A. flavus* and *A. parasiticus* populations [53,54]. Indeed, the ratio 1:1 is not discernable due to the few strains isolated and curated in different collections.

The present study increases the number of species in the *A. flavus* clade, which is comprised of many heterothallic species that share common morphological characters, such as biseriate heads, greenish to brownish colony coloration, ability to produce sclerotia, among others [17]. Likewise, species in this clade are capable of producing aflatoxins, aspergillic acid, CPA, kojic acid, versicolorins, aflatrem, etc. [20,55]. Within the clade, the main difference between *A. flavus* and the remaining species (*A. minisclerotigenes*, *A. parvisclerotigenus* and *A. korhogoensis* sp. nov.), is that *A. flavus* has lost the ability to produce G-aflatoxins [22]. Another important difference is that *A. flavus* is comprised of two morphotypes: small sclerotium producers and those that are able to produce larger sclerotia than the other three species. *Aspergillus flavus* is a ubiquitous fungus, being readily sampled across the globe, but *A. minisclerotigenes*, *A. parvisclerotigenus,* and *A. korhogoensis* sp. nov. have smaller geographic distributions. For example, *A. minisclerotigenes* has been isolated from Africa, South and North America, Europe, and Australia, whereas *A. parvisclerotigenus* has been isolated from Guinea Gulf [16], and *A. korhogoensis* has only been found in the Côte d’Ivoire. 

Herein, we proposed that *Aspergillus korhogoensis* sp. nov. is the sister taxon of *A. parvisclerotigenus*, based on secondary metabolite analyses, morphology and molecular evidence. Both species share a similar secondary metabolic profile according to Frisvad et al. [9]. However, there are some differences in their secondary metabolite production. Unlike *A. parvisclerotigenus*, the production of A-30461 (aspirochlorin) was not observed in any *A. korhogoensis* extracts. On the other hand, *A. korhogoensis* produced aflavinines, asparasones and leporin B. A pattern close to each other could be appreciated while comparing morphological characters, though subtle differences were observed between strains of both species. On AFPA, *A. parvisclerotigenus* had a trend to produce yellowish spores and highly floccose colonies, whereas *A. korhogoensis* sp. nov. tended to have flatter colonies and reduced sporulation. At the molecular level, the concatenation of nine different loci strongly suggested that they were two different species. The inclusion of strains from different populations of *A. parvisclerotigenus* (Benin, Nigeria and Côte d’Ivoire) reduced the possibility of any artifact linked with polymorphisms. 

Sub-Saharan West Africa, and especially the Guinea Gulf, displays an interesting diversity of species from section *Flavi*, including several cryptic species, although *A. flavus* continues to be the most frequent species sampled [56]. Despite the prevalence of *A. flavus*, it is noteworthy that other S-strain species are present at lower rates, are usually G-aflatoxin producers, and their production of aflatoxins is usually higher than that of *A. flavus* sensu stricto [57,58]. Some strains, previously characterized as *A. flavus* S_BG_, are nowadays being classified as *A. minisclerotigenes*, *A. parvisclerotigenus*, and in this study as *A. korhogoensis*. In different countries of the Guinea Gulf, strains exhibiting the S_BG_ chemotype have been associated with drier agroecological zones bordering the Sahara desert [14,59,60]. Cadwell and Cotty [59] suggested that production of G-aflatoxins in Northern Benin could be mainly due to S_BG_ strains. Likewise, in this area, the environmental conditions could allow the presence of species that could be more sensitive to climate changes, resulting in a shift of the frequency of these strains [59]. Inter-specific sex is possible for these fungi, which has been shown via laboratory crosses [51]. All that is required for these strains to override heterokaryon incompatibility, or even species boundaries, is the need to circumvent an unfavorable environmental situation [61]. It may eventually be determined that many of the recently characterized novel species, with such similar morphological, genetic, metabolic and physiological characteristics, are hybrids resulting from cryptic inter-specific sex that comprise a species complex. However, much more research is required before this can be proven or refuted. Moore and co-workers [62,63,64] are sequencing the genomes of aflatoxigenic fungi in an effort to determine the relatedness of these fungi and to elucidate the evolution of aflatoxin production. The comparison of the genomes of species close to *A. flavus* such as *A. minisclerotigenes*, *A. parvisclerotigenus* and *A. korhogoensis* will also help to understand the genetic determinants of the *A. flavus* success.

## 4. Materials and Methods 

### 4.1. Chemicals

Solvents (phenol, chloroform, ethanol, ethyl acetate, methanol, and acetonitrile) of analytical grade used in the extraction and high-performance liquid chromatography (HPLC) were obtained from ThermoFisher Scientific (Illkirch, France). Ultrapure water used for HPLC with Diode Array Detector (DAD), LC/MS analyses, and for molecular biology experiments was purified from a MilliQ purification system (Millipore, Billerica, MA, USA). Unless otherwise specified, chemicals were purchased from Sigma-Aldrich (Saint Quentin Fallavier, France). 

### 4.2. Fungal Isolates and Culture Conditions

Seven atypical S_BG_ *A. flavus* strains were isolated in 2014 from peanuts in the Northern Korhogo region of Côte d’Ivoire. Four other atypical S_BG_ strains isolated from food, decaying leaves, and logs of wood collected in Southwest Nigeria [65] were added. To identify these eleven S_BG_ strains to species level, we compared them against a dataset comprised of strains obtained from different international collections and stored under controlled conditions at Research Center in Food Toxicology TOXALIM, Toulouse. We included at least one strain belonging to most species within the section *Flavi* (Table 2). The isolates were cultured on Malt Extract Agar (MEA) (Biokar Diagnostics, Allone, France) at 25 °C for seven days, and stored as spore suspensions on 20% glycerol for further analyses. The *A. korhogoensis* Type strain, along with three other strains, were deposited at Agricultural Research Service Culture Collection (NRRL) (Peoria, IL, USA).

### 4.3. DNA Extraction and Amplification

A loopful of spores from each of the examined *Aspergillus* isolates was inoculated on Yeast Extract Sucrose (YES) liquid medium, and kept in agitation in an orbital incubator at 170 rpm at 27 °C for five days. DNA extraction was performed according to Girardin et al. [71] by grinding a portion of mycelium in a 5 mL mortar on ice, followed by the addition of 5.5 mL lysis buffer 2 (5 mL Tris-HCL 1 M, 3.65 g NaCl, 12.5 mL EDTA 0.5 M pH 8, 2.5 g SDS, H_2_O qsp 250 mL). The content was transferred to a 15 mL tube, and 12.5 µL of proteinase K were added, before being incubated from 30 min to 1 h at 37 °C, and then incubated for 10 min at 65 °C. Afterwards, one volume of phenol/chloroform (7:3, *v*/*v*) was added and samples were then centrifuged at 3000× *g* for 1 h. The supernatant was recovered into a new tube, where 6 µL RNAse were added, and it was subsequently incubated for 2–3 h at 37 °C. Next, one volume of chloroform was added and centrifuged at 3000× *g* for 10 min. The supernatant was recovered into a new tube and one volume of isopropanol was added. At this point, samples were softly shaken for 2 h in a horizontal shaker and kept overnight at 4 °C. The following day, samples were centrifuged at 10,000× *g* for 30 min. The supernatant was eliminated and the pellet carefully washed with 300 µL of 70% ethanol, and centrifuged at 10,000× *g* for 15 min, followed by a gentle aspiration of the supernatant. Finally, the pellet was resuspended with 30 µL of pure water. DNA of samples was quantified using a NanoDrop ND-1000 (NanoDrop Technologies, Wilmington, DE, USA).

### 4.4. Amplification and Sequencing of Genomic Loci

Genes were amplified as follows: (1) pre-denaturation at 94 °C for 5 min; (2) denaturation at 94 °C for 45 s; (3) annealing at 55–57.3 °C for 1 min (55 °C = *ITS, benA, cmdA, mcm7*, *preB* and *preA*; 56 °C = *ppgA*; 57.3 °C = a*mdS* and *rpb1*); (4) extension at 72 °C for 1 min (Steps 2 to 4 were carried out for 40 cycles); (5) final extension at 72 °C for 10 min; and (6) final temperature hold at 4 °C. Primers used in the study are shown in Table S1. Polymerase Chain Reaction (PCR) amplifications were performed in a C1000 Touch^TM^ thermal cycler (BioRad, Marnes-la-Coquette, France). PCR amplicons were purified with GeneElute^TM^ PCR Clean-Up Kit (Sigma-Aldrich, Saint Quentin Fallavier, France). Sanger sequences were obtained by using the Applied Biosystems Big Dye Terminator v3.1 chemistry (ThermoFisher Scientific, Illkirch, France), they were then purified with the Applied Biosystems Big Dye XTerminator protocol (Thermofisher Scientific, Illkirch, France) and finally processed on the ABI 3130xl Genetic Analyzer (Thermofisher Scientific, Illkirch, France), available on the GeT-Purpan technological facility (Genome and Transcriptome, GenoToul, Toulouse, France). New sequences were deposited in GenBank and accession numbers are reported in Table S2. Sequence data of some isolates, obtained from previously accessioned data in the GenBank database, were included for constructing phylogenetic trees (Table S2). 

### 4.5. Alignment, Model Selection, and Molecular Analyses

Data were assembled, aligned and trimmed in BioEdit/ClustalW (http://www.mbio.ncsu.edu/bioedit/bioedit.html). Gene regions with multiple gaps were aligned to minimize indels and optimize nucleotide identities among different strains. Sequences from multiple genomic regions were concatenated using Mesquite v3.2 [72], but the mating type loci were analyzed independently. For concatenated data, the best-fit nucleotide substitution models and partitioning scheme were chosen using PartitionFinder v2.0.0 [73] under BIC. To search for the best-fit scheme, a greedy algorithm with linked branch lengths of alternative partitions was used. Partitions obtained consisted of four subsets that corresponded to a specific model (noted in parentheses): Subset 1 included *ppgA*, *cmdA, benA*, *rpb1* and *mcm7 =* 2129 bp (K80+G); Subset 2 included *ITS* = 778 bp (TRNEF+I); Subset 3 included *preB* and *preA* = 1223 bp (HKY+G); and Subset 4 included *amdS* = 491 bp (K80+G).

Bayesian inference statistical methods were used to obtain tree topologies for concatenated data, using the best-fit substitution models listed above. For Bayesian analyses, MrBayes v3.2 [74] was used, and four independent runs were carried out for 10^7^ generations, each with four chains, Markov Chain Monte Carlo, and sampling every 10^3^ generations. We confirmed for each analysis that the average standard deviation of split frequencies between chains approached values of ≤0.01, and the potential scale factor reduction factor (PSRF) to 1. For all the analyses, and from the total number of trees per run, 25% were arbitrarily discarded as “burn-in”. The remaining trees were used to calculate posterior probabilities (PP) for each bipartition in a 50% majority-rule consensus tree using Tracer v1.6 [75]. Phylogenetic trees were visualized and edited with FigTree v1.4.2 [76]. 

### 4.6. Morphological and Physiological Studies

Morphological and growth analyses were carried on MEA, High Salt MEA (MS) (MEA complemented with NaCl 60 g/L), Czapek agar (CZ) (Oxoid, Dardly, France), Czapek Yeast Autolysate agar (CYA), YES agar. Physiological analyses were carried out on creatine agar (CREA) [77], and *Aspergillus flavus*/*parasiticus* agar (AFPA) [78]. Strains MACI46 (NRRL 66708), MACI219 (NRRL 66709), MACI254 (NRRL 66710), MACI264 (NRRL 66711), *A. parvisclerotigenus* CBS 121.62 and *A. minisclerotigenes* J117e were used for the analyses on MS, CYA, CZ, YES, and CREA. In addition, four strains belonging to *A. parvisclerotigenus* were included for the analyses on MEA and AFPA: MACI8, MACI258, MACI221, and AFc36. 

Cultures on MEA, MS, CZ, CYA, YES, CREA, and AFPA were seeded with three calibrated inoculates of 500 spores, and incubated in the dark at 25 °C for seven and ten days. Macroscopic characters were observed with a stereomicroscope SZX9—X12-120 (Olympus, Rungis, France). Microscopic characters were observed on MEA at 7 and 10 days using a microscope CX41—X400 and X1000 (Olympus, Rungis, France). In addition, growth analyses were calculated from MEA cultures, which were centrally inoculated with 10^3^ spores, and incubated at 25 °C, 37 °C and 42 °C for seven days [17,79,80].

### 4.7. LC/MS Secondary Metabolic Characterization

#### 4.7.1. Secondary Metabolic Characterization of Whole Fungal Culture 

Pre-cultures of strains MACI46 (NRRL 66708), MACI219 (NRRL 66709), MACI254 (NRRL 66710) and MACI264 (NRRL 66711) incubated in the dark at 27 °C for seven days. For metabolite profile characterization, isolates were cultured in four different media: MEA, CYA, YES agar, and Potato Dextrose Agar medium (PDA) (Sigma-Aldrich, Saint Quentin Fallavier, France). For each medium, three biological replicates were inoculated centrally with 10 µL of calibrated spore suspensions (10^5^ spores/mL) prepared from seven-day cultures on 7.5 cm Petri dishes. The samples were incubated in the dark at 27 °C for seven days. 

To perform extrolite extractions, culture media were macerated and placed in 50 mL sterilized tubes, and thereafter 35 mL of ethyl acetate was added to each sample. Samples were agitated 48 h in an orbital incubator at 170 rpm at room temperature. Ethyl acetate was filtered through a Whatman 1PS phase separator (GE Healthcare Life Sciences, Vélizy-Villacoublay, France) and evaporated at 60 °C until dry. Samples were then dissolved in 400 µL of methanol. To eliminate possible impurities, each sample was filtered through a 0.45 µm disk filter (ThermoFisher Scientific, Illkirch, France) [81]. 

#### 4.7.2. Secondary Metabolic Characterization of Sclerotia 

The isolates were cultured on MEA whereby a loopful of spores was taken from seven-day cultures and streaked onto 9 cm Petri dishes. The MEA samples were incubated in the dark at 27 °C for eight days. To recover sclerotia from culture media, 10 mL of 0.01% Triton-X solution were added to each Petri dish. Sclerotia were gently scraped and transferred into 15 mL tubes. To remove mycelium and conidium debris, 10 mL of 0.01% Triton-X were added to each tube and homogenized in a vortex. Once the sclerotia precipitated, the supernatant was discarded. This step was carried out 4 to 5 times to eliminate possible residual debris [82]. 

Sclerotia were transferred into a 5 mL mortar. Then 5 mL of ethyl acetate were gently added while grinding the sclerotia. This step was carried out three times. Next, 5 mL of chloroform were gently added and the same procedure followed. This step was repeated three times. Samples were evaporated at 60 °C until dry, and resuspended in 0.5 mL solution methanol/acetonitrile/H_2_O qsp (30:30:40, *v*/*v*/*v*) [31,82]. To remove possible impurities, samples were filtered through 0.45 µm disk filters (ThermoFisher Scientific, Illkirch, France).

#### 4.7.3. Secondary Metabolic Analysis 

Extrolite analyses were carried out on a HPLC apparatus coupled to an LTQ Orbitrap XL high-resolution mass spectrometer (HRMS) (ThermoFisher Scientific, Illkirch, France). Extracted samples contained 10 µL of each replicate diluted with 170 µL methanol. For the analyses, 10 µL of each sample were injected into a reverse-phase 5 µm Luna C18 column (150 mm × 2.0 mm) (Phenomenex, Torrance, CA, USA) operated at a flow rate of 0.2 mL/min. A gradient program of 0.1% formic acid in water (phase A) and 100% acetonitrile (phase B) was executed as follows: 0 min 20% B, 30 min 50% B, from 35 to 45 min 90% B, from 50 to 60 min 20% B. HRMS acquisitions were obtained by electrospray ionization (ESI) in the positive and negative modes under the subsequent parameters: (1) positive mode: spray voltage + 4.5 kV, capillary temperature 350 °C, sheath gas (N_2_) flow rate 40 au (arbitrary units), auxiliary gas (N_2_) flow rate 6 au; and (2) negative mode: spray voltage—3.7 kV, capillary temperature 350 °C, sheath gas (N_2_) flow rate 30 au, auxiliary gas (N_2_) flow rate 10 au. Full MS spectra were accomplished at a resolution of 60,000 with a mass-to-charge ratio (*m*/*z*) range 50–800. The MS/MS spectra were generated by collision-induced dissociation (CID) according the following parameters: collision energy = 35 eV, resolution = 7500, isolation width = 1.5 Da, activation Q = 0.250, and activation time = 30 ms.

## Figures and Tables

**Table 1:** Principal secondary metabolites produced by *Aspergillus korhogoensis*.

**Metabolite**	**Elemental Composition**	***m*/*z***	**Ion**	**Retention Time (min)**	**MS/MS**	**Error (ppm)**	ID Level *	**References**
AFLATOXIN BIOSYNTHESIS PATHWAY								
Aflatoxin B_1_	C_17_H_12_O_6_	313.07	[M + H]^+^	17.37	285 (100), 298, 284, 270, 257, 243, 229	−0.398	1, 2	[25]
Aflatoxin B_2_	C_17_H_14_O_6_	315.07	[M + H]^+^	14.95	297, 287 (100), 259, 269, 273	−5.920	1, 2	[25]
Aflatoxin G_1_	C_17_H_12_O_7_	329.08	[M + H]^+^	15.25	311 (100), 301, 300, 283, 243	−0.119	1, 2	[25]
Aflatoxin G_2_	C_17_H_14_O_7_	331.08	[M + H]^+^	12.84	313 (100), 303, 285, 275, 257, 245	−0.511	1, 2	[25]
*O*-methyl-sterigmatocystin	C_19_H_14_O_6_	339.08	[M + H]^+^	24.21	324 (100), 311, 306, 295	2.817	1	
Sterigmatocystin	C_18_H_12_O_6_	325.07	[M + H]^+^	33.59	310 (100), 297, 282	0.570	1, 2	[26]
Versicolorin A	C_18_H_10_O_7_	337.03	[M − H]^−^	35.95	309 (100), 319, 308, 293, 265, 253	−2.094	1, 2	[27]
Versicolorin B	C_18_H_12_O_7_	339.05	[M − H]^−^	34.40	311 (100) 310, 309, 295, 297, 283	−0.578	1, 2	[27]
Norsolorinic acid	C_20_H_18_O_7_	369.10	[M − H]^−^	42.07	351 (100), 341, 325, 308, 297, 270	1.528	1	
CYCLOPIAZONIC ACID BIOSYNTHETIC PATHWAY								
α-cyclopiazonic acid	C_20_H_20_N_2_O_3_	337.15	[M + H]^+^	36.77	182 (100), 196, 154, 140	0.561	1, 2	[28]
β-cyclopiazonic acid	C_20_H_22_N_2_O_3_	339.17	[M + H]^+^	37.58	198 (100), 324, 283, 183, 144, 130	−1.289	2	[28]
2′-oxo-cyclopiazonic acid	C_20_H_20_N_2_O_4_	353.15	[M + H]^+^	36.20	335 (100), 311, 293, 252, 224, 212	−1.174	2	[28]
3′-hydroxy-speradine A	C_21_H_22_ N_2_O_5_	383.16	[M + H]^+^	21.19	355 (100), 365, 182, 184, 226, 254, 323, 347, 337	−1.144	2	[28]
Speradine C	C_20_H_22_ N_2_O_5_	371.16	[M + H]^+^	18.19	353 (100), 287, 269, 259, 226, 184	2.780	2	[28]
Speradine D	C_20_H_22_ N_2_O_6_	387.16	[M + H]^+^	20.80	369 (100), 269, 226, 184	2.679	2	[28]
Speradine F	C_21_H_22_ N_2_O_7_	415.15	[M + H]^+^	18.99	397 (100), 379, 369, 355, 353, 337, 311, 297, 281, 269, 253, 226, 184	−0.644	2	[28]
Cyclopiamide J	C_22_H_24_N_2_O_7_	429.17	[M + H]^+^	23.96	287 (100), 411, 497, 379, 369, 337, 269, 259, 226, 184	−0.693	2	[28]
KOJIC ACID BIOSYNTHETIC PATHWAY								
Kojic acid	C_6_H_6_O_4_	143.03	[M + H]^+^	1.87	143 (100) 125, 113, 97	1.432	1, 2	[29]
AFLATREM BIOSYNTHETIC PATHWAY								
α-aflatrem	C_32_H_39_NO_4_	502.29	[M + H]^+^	41.45	444 (100), 484, 426, 412, 376, 198	1.144		
Paspalinine	C_27_H_31_NO_4_	434.23	[M + H]^+^	39.22	376 (100), 416, 419, 362, 358, 344, 130	0.726	2	[30]
Paspaline	C_28_H_39_NO_2_	422.31	[M + H]^+^	43.96	130 (100), 404, 407	−0.583	2	[30]
Hydroxyaflatrem	C_32_H_39_NO_5_	518.29	[M + H]^+^	38.22	460 (100), 500, 482, 442, 446, 428	−0.347		
Paxilline	C_27_H_33_NO_4_	436.25	[M + H]^+^	38.64	418 (100), 421, 400, 378, 360, 346, 130	−2.762	1, 2	[30]
13′-desoxypaxilline	C_27_H_33_NO_3_	420.25	[M + H]^+^	40.31	402 (100), 405, 362, 130	−0.320	2	[30]
ASPARASONE BIOSYNTHESIS PATHWAY								
Asparasone A	C_18_H_14_O_8_	357.06	[M − H]^−^	22.13	339 (100) 299	1.315	2	[31]
1,3,4,6,8 pentahydroxy-2-(1′-hydroxy-3′-oxobuty)anthraquinone	C_18_H_14_O_9_	373.04	[M − H]^−^	9.36	355 (100) 315	0.629	2	[31]
1,3,6,8 tetrahydroxy-2-(1′-hydroxyethyl) anthraquinone	C_16_H_12_0_7_	315.05	[M − H]^−^	27.98	297 (100)	0.775	2	[31]
1,3,6,8 tetrahydroxy-2-(3′ oxobut 1′-en-1′-yl) anthraquinone	C_18_H_12_0_7_	339.05	[M − H]^−^	29.77	297 (100) 321, 296, 295, 311, 306	1.428	2	[31]
LEPORINS BIOSYNTHESIS PATHWAY								
Leporin B	C_22_H_25_NO_3_	352.19	[M + H]^+^	40.78	216 (100), 230, 244, 258, 270, 282, 296, 306	−1.505	2	[25]
Leporin B precursor	C_22_H_25_NO_2_	336.20	[M + H]^+^	37.97	200 (100), 214, 228, 242, 254, 266, 280	0.102	2	[25]
AFLAVARIN BIOSYNTHESIS PATHWAY								
Aflavarin	C_24_H_22_O_9_	455.13	[M + H]^+^	18.22	413 (100), 425, 437, 395, 379, 364, 348, 303	−3.732	1, 2	[32]
7′-demethyl-siderin	C_11_H_10_O_4_	207.07	[M + H]^+^	13.58	163 (100), 177, 175, 148, 147, 135, 133, 131, 115, 107	0.312	2	[32]
Aflavarin precursor 6	C_22_H_18_O_8_	411.11	[M + H]^+^	20.69	369 (100), 381, 379, 352, 343, 337, 279, 207, 177, 147	−0.569	2	[32]
Aflavarin precursor 5	C_23_H_20_O_8_	425.12	[M + H]^+^	26.75	383 (100), 393, 369, 363, 357, 349	0.484	2	[32]
Aflavarin precursor 4	C_24_H_22_O_8_	439.14	[M + H]^+^	30.52	397 (100), 383, 371, 367, 365, 351, 341, 321	−0.624	2	[32]
AFLAVININE BIOSYNTHESIS PATHWAY								
20′-hydroxyaflavinine	C_28_H_39_O_2_N	404.29	[M − H_2_O + H]^+^	37.53	386 (100), 287, 269, 243, 144, 130	0.071	1	
Unknown aflavanine	C_28_H_39_O_2_N	404.29	[M − H_2_O + H]^+^	38.14	386 (100), 287, 269, 224	0.170		

* ID Level 1: Metabolites that displayed the same retention time, UV and MS/MS spectra than the authentic standard. Level 2: Metabolites that displayed the same UV spectrum and/or the same MS/MS fragmentation pattern in accordance with the literature.

**Table 2:** *Aspergillus* isolates used in this study.

**Strain**	**Sampling Data**		**Reference**
	**Substrate**	**Country**	
***A. arachidicola***			
CBS 117610^T^ = IBT 25020	*Arachis glabatra* leaf	Argentina	[20]
CBS 117614 = IBT 27183	*Arachis glabatra* leaf	Argentina	[20]
***A. bertholletius***			
CCT 7615^T^	Soil near *Bertholletia excelsa* trees	Brazil	[19]
***A. bombycis***			
NRRL 26010^T^ = CBS 117187	Frass, silkworm rearing house	Japan	[66]
***A. caelatus***			
NRRL 25528^T^ = ATCC 201128 = CBS 763.97 = JCM 10151	Peanut field soil	Georgia, USA	Horn B.W., National Peanut Lab, Dawson, GA (in NRRL database)
***A. flavus***			
NRRL 3518	Wheat flour	Illinois, USA	Graves NRRL isolate (in NRRL database)
NRRL 4818 = CBS 16870	Food, butter	USA	Fennell D.I., University of Wisconsin, Madison, Wisconsin (in NRRL database)
NRRL 3357 = CBS 128202	Peanut cotyledons	USA	[67]
***A. minisclerotigenes***			
CBS 117635^T^	*Arachis hypogaea* seed	Argentina	[20]
NRRL 29000	Peanut soil	Australia	Geiser D., Pennsylvania State University (in [21])
E21	Cumin	Morocco	[15]
E44	White pepper	Morocco	[15]
E74	Paprika	Morocco	[15]
***A. mottae***			
MUM 10.231^T^ = CBS 130016	Maize seed	Portugal	[18]
***A. nomius***			
NRRL 13137^T^ = CBS 260.88	Wheat	Illinois, USA	Schindler A.F., FDA, Washington D.C. (in NRRL database)
***A. novoparasiticus***			
CBS 126849^T^ = LEMI 250	Sputum, leukemic patient	São Paulo, Brazil	[23]
LEMI 149	Hospital air	São Paulo, Brazil	[23]
LEMI 267	Sputum, leukemic patient	São Paulo, Brazil	[23]
AFc31 = NRRL 62794	Cassava	Benin	[14]
AFc32 = NRRL 62795	Cassava	Benin	[14]
***A. oryzae***			
CBS 100925^T^ = IMI 16266 = NRRL 447	Unknown source	Japan	[17]
RIB40	Cereal (broad bean)	Kyoto, Japan	[68]
***A. parasiticus***			
CBS 100926^T^	*Pseudococcus calceolariae*, sugar cane mealy bug	Hawaii, USA	[17]
NRRL 492	Unknown source	China	[23]
***A. parvisclerotigenus***			
CBS 121.62^T^	*Arachis hypogea*	Nigeria	[9]
AFc36 = NRRL 62796	Cassava	Benin	[14]
MACI8	Peanuts	Côte d’Ivoire	This study
MACI221	Peanuts	Côte d’Ivoire	This study
MACI258	Peanuts	Côte d’Ivoire	This study
SF1	Rain forest soil	Nigeria	[65]
SF3	Rain forest soil	Nigeria	[65]
SF6	Rain forest soil	Nigeria	[65]
SF9	Food item	Nigeria	[65]
***A. pseudocaelatus***			
CBS 117616^T^	*Arachis burkartii* leaf	Argentina	[17]
***A. pseudotamarii***			
NRRL 443	Soil	Brazil	[69]
NRRL 25518	Tea field soil	Miyazaki, Japan	[70]
***A. sergii***			
MUM 10.219^T^ = CBS 130017	Almond shell	Portugal	[18]
***A. sojae***			
CBS 100928^T^	Soy sauce	Japan	[17]
***A. transmontanensis***			
MUM 10.214^T^ = CBS 130015	Almond shell	Portugal	[18]
***A. korhogoensis*** **sp. nov.**			
MACI254^T^	Peanuts	Côte d’Ivoire	This study
MACI46	Peanuts	Côte d’Ivoire	This study
MACI219	Peanuts	Côte d’Ivoire	This study
MACI264	Peanuts	Côte d’Ivoire	This study

CBS, Centraalbureau voor Schimmelcultures, Utrecht, The Netherlands; NRRL: National Center for Agricultural Utilization Research, U.S. Department of Agriculture, Peoria, IL, USA; LEMI: Laboratório Especial de Micologia, São Paulo, Brazil; MUM: Micoteca da Universidade de Minho, Braga, Portugal; CCT: Coleção de Cultura Tropical, Campinas, Brazil; SF: Southern Regional Research Center, U.S. Department of Agriculture, New Orleans, USA.

## Supplementary Materials

The following are available online at www.mdpi.com/2072-6651/9/11/353/s1, Table S1: Primers used to amplify multiple genomic regions within *Aspergillus* species, Table S2: Isolates examined and accession numbers deposited in GenBank.
