# Supplementary material for: Aspergillus korhogoensis, a Novel Aflatoxin Producing Species from the Côte d’Ivoire
Source: Toxins (Basel). 2017 Oct 31;9(11):353. doi: 10.3390/toxins9110353 (PMC5705968; doi:10.3390/toxins9110353)
Supplement: Supplementary file 1 [file toxins-09-00353-s001.pdf]

Supplementary Materials: *Aspergillus korhogoensis*, a Novel Aflatoxin Producing Species from the Côte d'Ivoire

Amaranta Carvajal-Campos, Ama Lethicia Manizan, Souria Tadrist, David Koffi Akaki, Rose Koffi-Nevry, Geromy G. Moore, Stephen O. Fapohunda, Sylviane Bailly, Didier Montet, Isabelle P. Oswald, Sophie Lorber, Catherine Brabet and Olivier Puel

Table S1. Primers used to amplify multiple genomic regions within *Aspergillus* species.

Gene	Length bp*	Primers	Sequence	Reference
ITS	1152	Its1	5' – GGAAGTAAAGTCGTAACAAGG-3′	[1]
		D2r	5' – TTGGTCCGTGTTTCAAGACG-3′	[2]
benA	541	Btub2a	5' – GGTAACCAAATCGGTGCTGCTTTC-3′	[3]
		Btub2b	5' – ACCCTCAGTGTAGTGACCCTTGGC-3′	[3]
cmdA	543	cmd5	5' – CCGAGTACAAGGAGGCCTTC-3′	[4]
		cmd6	5' – CCGATAGAGGTCATAACGTGG-3′	[4]
rpb1	860	FORWARD	5' – GARTGYCCDGGDCAYTTYGG-3′	This study
		REVERSE	5' – CCNGCDATNTCRTTRTCCATRTA-3′	This study
mcm7	544	mcmF	5' – CAATGCCTACACTTGTGATCGC-3′	This study
		mcmR	5' – CTCCAATGAGCAAAAGAAGCAAG-3′	This study
amdS	566	amdS1	5' – CCATCGGTATAGGAACTGA-3′	[5]
		amdS2	5' – AGGGTGCCACGGTATGTC-3′	[5]
Mat1-1	241	M1F	5' – ATTGCCCATTTGGCCTTGAA-3′	[6]
		MAT1R3*	5' –ACMGARTARTTGGTMGAAATATCGGCTTC-3′	This study
Mat1-2	645	MAT2F2*	5' – GAYGCTYTGCGTCACCTYGAG-3′	This study
		M2R	5' – GCTTCTTTTCGGATGGCTTGCG-3′	[6]
preA	1336	PreAF2	5' – TGCTSACCATCMCTCCSTTGATCTT-3'	This study
		PreAR3	5' - GCTNGTNCCTGCCCATGCATTWG-3′	This study
preB	777	PreBF1	5' – ATCCAGATCTGCATCAACT-3′	This study
		PreBR1	5' – AGCGGGAGAGAGATAGTGACCAG-3′	This study
ppgA	258	ppgAF	5' – GCAGCCACCAGTGTACAGGC-3′	This study
		ppgAR	5' – CCATAGCATCCGCAAGGGCATC-3′	This study

* Amplicon size corresponds to *A. flavus* NRRL3357 sequences except for *Mat1-2*. For *Mat1-2,* amplicon size corresponds to *A. bombycis* sequence.

Table S2: Isolates examined and accession numbers deposited in GenBank. In bold sequences recovered from GenBank; normal sequences obtain in this study. T = type strain, * = strains of which the whole genome is sequenced; ** = also found under accession number JX456214; / = gene not present.

Table continues in following pages

SPECIES AND ISOLATE NUMBER	ACCESSION NUMBER										
	ITS	*benA*	*cmdA*	*mcm7*	*amdS*	*rpb1*	*preB*	*ppgA*	*preA*	*Mat1-1*	*Mat1-2*
A. arachidicola											
CBS 117610T	MF668184	**EF203158**	**EF202049**	MF427568	**GU203491**	MF448475	MF427533	MF427635	MF427600	MF966969	/
CBS 117614	KY937923	KY924665	KY924677	MF427569	MF427670	MF448476	MF427534	MF427636	MF427601	/	MF448470
A. bertholletius											
CCT 7615T	KY937924	KY924666	KY924678	MF427570	MF427671	MF448477	MF427535	MF427637	MF427602	MF448449	/
A. bombycis											
NRRL 26010T	**AF104444**	**AY017547**	**AY017594**	**JQ690064**	MF427672	MF448478	**OGM45283**	**OGM40854**	**OGM42599**	/	**OGM45987**
A. caelatus											
NRRL 25528T	**AF004930**	**EF661470**	**EF661522**	**JQ690063**	MF427673	MF448479	MF427536	MF427638	MF427603	MF448450	/
A. flavus											
NRRL 3518	**EF661552**	**EF661487**	**EF661510**	MF427572	MF427675	MF448481	MF427537	MF427640	MF427605	/	MF448471
NRRL 4818	**EF661563**	**EF661489**	**EF661510**	MF427571	MF427674	MF448480	MF427538	MF427639	MF427604	MF448451	/
NRRL 3357*	MF966967	**M38265**	**EED55330**	**EED52746**	**EED48415**	**EED56055**	**EED51811**	**EED56518**	**EED51899**	**EED46656**	/
A. minisclerotigenes											
CBS 117635T	KY937925	KY924667	KY924679	MF427573	MF427677	MF448483	MF427539	MF427642	MF427607	MF448453	/
E21	KY937926	**JX456195**	**JX456196**	MF427574	MF427678	MF448484	MF427540	MF427643	MF427608	**JX456194**	/
E44	KY937927	**JX456210**	**JX456214**	MF427575**	MF427679	MF448485	MF427541	MF427644	MF427609	/	**JX456216**
E74	KY937928	**JX456211**	**JX456212**	MF427576	MF427680	MF448486	MF427542	MF427645	MF427610	/	**JX456213**
NRRL 29000	KY937929	KY924668	KY924680	MF427577	MF427676	MF448482	MF427543	MF427641	MF427606	MF448452	/
A. mottae											
MUM 10.231T	**JF412768**	**HM803086**	**HM803015**	**HM803059**	MF427681	MF448487	MF427544	MF427646	MF427611	**HM803042**	/

SPECIES AND ISOLATE NUMBER	ACCESSION NUMBER										
	ITS	*benA*	*cmdA*	*mcm7*	*amdS*	*rpb1*	*preB*	*ppgA*	*preA*	*Mat1-1*	*Mat1-2*
A. novoparasiticus											
CBS 126849^{T}	KY937930	KY924673	KY924685	MF427583	**GU203478**	MF448490	MF427549	MF427649	MF427614	MF448456	/
AFc31	**KC964099**	KY924669	KY924681	MF427579	**KC921994**	MF448488	MF427545	MF427647	MF427612	MF448454	/
AFc32	**KC964100**	KY924670	KY924682	MF427580	**KC921995**	MF448489	MF427546	MF427648	MF427613	MF448455	/
LEMI 149	KY937931	KY924671	KY924683	MF427581	MF427682	MF448491	MF427547	MF427650	MF427615	MF448457	/
LEMI 267	KY937932	KY924672	KY924684	MF427582	**GU203480**	MF448492	MF427548	MF427651	MF427616	MF448458	/
A. oryzae											
CBS 100925^{T}	MF668185	**EF203138**	**EF202055**	MF427584	MF427683	MF448493	MF427550	MF427652	MF427617	MF448459	/
RIB40*	**AP007173**	**BAE64122**	**XP_001820302**	**BAE65095**	**D10492**	**XP_001819667**	**BAE62296**	**AP007155**	**XP_001823505**	**BAE63328**	/
A. parasiticus											
CBS 100926^{T}	KY937933	**EF203155**	**EF202043**	MF427585	**GU203493**	MF448494	MF427551	MF427653	MF427618	/	MF448472
NRRL 492	KY937934	KY924674	KY924686	MF427586	**GU203494**	MF448495	MF427552	MF427654	MF427619		MF495344
A. parvisclerotigenus											
CBS 121.62^{T}	MF668183	**EF203130**	**EF202077**	MF427587	MF427684	MF448496	MF427553	MF427655	MF427620	MF448460	/
AFc36	**KC964102**	**KC954604**	**KC954606**	MF427588	MF427685	MF448497	MF427554	MF427656	MF427621	MF448461	/
MACI8	KY689163	KY628794	KY661270	MF427589	MF427686	MF448498	MF427555	MF427657	MF427622	MF448462	/
MACI221	KY689198	KY628787	KY661291	MF427590	MF427687	MF448499	MF427556	MF427658	MF427623	MF495342	/
MACI258	KY689205	KY628789	KY661293	MF427591	MF427688	MF448500	MF427557	MF427659	MF427624	MF495343	/
SF1	MF668179	MF521634	MF521638	MF521646	MF521642	MF521662	MF521658	MF521650	MF521654	MF537434	/
SF3	MF668180	MF521635	MF521639	MF521647	MF521643	MF521663	MF521659	MF521651	MF521655	MF537435	/
SF6	MF668181	MF521636	MF521640	MF521648	MF521644	MF521664	MF521660	MF521652	MF521656	MF537436	/
SF9	MF668182	MF521637	MF521641	MF521649	MF521645	MF521665	MF521661	MF521653	MF521657	MF537437	/
A. pseudocaelatus											
CBS 117616^{T}	KY937935	**EF203128**	**EF202037**	MF427592	MF427689	MF448501	MF427558	MF427660	MF427625	MF448463	/
A. pseudotamarii											
NRRL 443	**AF004931**	**EF661476**	**EF661520**	MF427593	MF427690	MF448502	MF427559	MF427661	MF427626	MF448464	/

SPECIES AND ISOLATE NUMBER	ACCESSION NUMBER										
	ITS	*benA*	*cmdA*	*mcm7*	*amdS*	*rpb1*	*preB*	*ppgA*	*preA*	*Mat1-1*	*Mat1-2*
NRRL 25518	KY937937	KY924675	KY924687	MF427594	MF427691	MF448503	MF427560	MF427662	MF427627	MF448465	/
A. sergii											
MUM 10.219^{T}	KY937936	**HM803082**	**HQ340097**	**HM803071**	MF427692	MF448504	MF427561	MF427663	MF427628	/	**HM802967**
A. sojae											
CBS 100928^{T}	MF668186	**KJ175494**	**KJ175550**	MF427595	**GU203490**	MF448505	MF427562	MF427664	MF427629	MF537438	/
A. transmontanensis											
MUM 10.214^{T}	**JF412771**	**HM803101**	**HM803020**	**HM803065**	MF427693	MF448506	MF427563	MF427665	MF427630	**HM803050**	/
A. korhogoensis spp. nov.											
MACI46	KY689207	KY628790	KY661265	MF427596	MF427694	MF448507	MF427564	MF427666	MF427631	MF448466	/
MACI219	KY689208	KY628791	KY661266	MF427597	MF427695	MF448508	MF427565	MF427667	MF427632	/	MF448474
MACI254^{T}	KY689209	KY628792	KY661267	MF427598	MF427696	MF448509	MF427566	MF427668	MF427633	MF448467	/
MACI264	KY689210	KY628793	KY661268	MF427599	MF427697	MF448510	MF427567	MF427669	MF427634	MF448468	/

CBS, Centraalbureau voor Schimmelcultures, Utrech, The Netherlands; NRRL: National Center for Agricultural Utilization Research, U.S. Department of Agriculture, Peoria, IL, USA; LEMI: Laboratório Especial de Micologia, São Paulo, Brazil; MUM: Micoteca da Universidade de Minho, Braga, Portugal; CCT: Coleção de Cultura Tropical, Campinas, Brazil. SF: Southern Regional Research Center, U.S. Department of Agriculture, New Orleans, USA.

References

1. White, T.J.; Bruns, T.; Lee, S.; Taylor, W. Amplification and direct sequencing of fungal ribosomal RNA genes for phylogenetics. In: Innis, M. (Ed.), PCR Protocols: a Guide to Methods and Applications. Academic Press, San Diego, 1990, pp. 315-322.
2. Peterson, S.M. Phylogenic analysis of *Aspergillus* species using DNA sequences from four loci. *Mycologia* **2008**, *100*, 205-226.
3. Glass, N.L.; Donaldson, G.C. Development of primer sets designed for use with the PCR to amplify conserved genes from filamentous ascomycetes. *Appl. Environ. Microbiol.* **1995**, *61*, 1323-30.
4. Hong, S.B.; Cho, H.S.; Shin, H.D.; Frisvad, J.C.; Samson, R.A. Novel *Neosartorya* species isolated from soil in Korea. *Int. J. Syst. Evol. Microbiol.* **2006**, *56*, 477-486.
5. Geiser, D.M.; Pitt, J.I.; Taylor, J.W. 1998. Cryptic speciation and recombination in the aflatoxin-producing fungus *Aspergillus flavus*. Proc. Nat. Acad. Sci. U. S. A. 95, 388-393.
6. Ramirez-Prado, J.H.; Moore, G.G.; Horn, B.W.; Carbone, I. Characterization and population analysis of the mating-type genes in *Aspergillus flavus* and *Aspergillus parasiticus*. *Fungal Genet. Biol.* **2008**, *45*, 1292-1299.
